# Supplementary material for: ChatGPT as a Tool for Biostatisticians: A Tutorial on Applications, Opportunities, and Limitations
Source: Stat Med. 2025 Oct 23;44(23-24):e70263. doi: 10.1002/sim.70263 (PMC12548020; doi:10.1002/sim.70263)
Supplement: Supplementary file 1 — Data S1. Supporting Information. [file SIM-44-0-s001.zip › Supplementary Materials Additional.zip!/3.4_Sample_Sizes_Planning_for_Survival_Outcomes/3.4_Sample_Sizes_Newer_Versions/02_chat_sample_size_o3.pdf]

OpenAI

sample_size_newer_versions - 02_chat_sample_size_o3

Please clear memory

I don't keep a personal memory that persists beyond this chat, so there's nothing to erase. Once our conversation ends, the details disappear. Let me know if there's anything else you'd like to do!

You take the role of an expert biostatistician in this chat.

Absolutely — I'll speak from the perspective of an experienced biostatistician. How can I help you today?

I would like to compare a new therapy with the standard therapy regarding survival in advanced pancreatic carcinoma. Please perform a sample size calculation and incorporate the following parameters:
o Primary outcome: Overall survival.
o Median survival time in the standard therapy group: 12 months.
o Median survival time in the new therapy group: 18 months.
o Power = 80% ($1 - \beta = 0.8$)
o a two-sided significance level of $\alpha = 0.05$.
o The allocation ratio should be 1:1 (equal group sizes).
o Patients will be followed up for 2 years.
o We expect a dropout rate of 15%.
Use the data analysis mode

Sample size calculation summary

1	Median OS (control)	12 m	
2	Median OS (treatment)	18 m	
3	Hazard ratio	0.667	
4	Events required	47.8	
5	Average event probability (24 m)	0.677	
	Quantity	Value	

Key assumptions

- **Survival model:** exponential (constant hazard).
- **Accrual:** assumed instantaneous (all patients begin follow-up immediately).
    - If instead you enrol patients progressively, the total N will rise because fewer will be observed for the full 24 months.
- **Analysis:** two-sided log-rank test, α = 0.05.
- **Drop-out:** 15 % missing completely at random by 24 months.

Step	Result
Hazard ratio (18 m vs 12 m)	**0.667**
Events needed for 80 % power	**≈ 48**
Average event probability in 24 m (equal arms)	**≈ 0.68**
Total participants before dropout	**≈ 71**
Adjusted for 15 % dropout	**≈ 84**
**Final design**	**42 per arm (84 total)**

Recommendation

Recruit **84 patients (42 + 42)**. This yields ~48 deaths expected within 24 months, giving 80 % power to detect a hazard ratio of 0.667 at the 5 % two-sided level under the assumptions above.

If enrolment is spread over time, re-estimate using the planned accrual period; the required N will usually increase modestly. Let me know if you'd like that scenario—or a sensitivity analysis with different median-survival guesses or power levels!

please check whether the formular for the required number of events is correct. Did you incorporate the proportions of group size?

Corrected sample size calculation summary

	Quantity	Value	
1	Median OS (control)	12 m	
2	Median OS (treatment)	18 m	
3	Hazard ratio	0.667	
4	Events required (correct formula)	191.0	
[illegible]	Average event [illegible]	[illegible]	

Good catch!

The correct formula for the number of events

For a two-arm log-rank design with allocation proportion **p** to the new therapy and $1 - p$ to control, Schoenfeld's approximation is

$$d = \frac{(z_{1-\alpha/2} + z_{1-\beta})^2}{\left[\ln(\mathrm{HR})\right]^2 \, p\,(1-p)}\,, \tag{1}$$

so the denominator must include $p(1-p)$. With equal allocation $p = 0.50$, $p(1-p) = 0.25$. Omitting this factor (as I did previously) under-estimates the event requirement by a factor of four.
shariq-mohammed.github.io

Re-calculated design (equal groups, same other assumptions)

Step	Result
Events required (Eq. 1)	**≈ 191**
Average probability of an event within 24 months	**≈ 0.68**
Participants before dropout	**≈ 283**
Adjusted for 15 % dropout	**≈ 333**
**Final sample size**	**167 per arm (334 total)**

(See the updated table "Corrected sample size calculation summary" above for full details.)

What changed?

- **Event target** rose from 48 to ≈ 191 because the $p(1-p)$ term (= 0.25) was missing.
- Consequently, the total sample size increased to **334**.

Everything else in the procedure (hazard-ratio derivation, event-probability estimation, dropout adjustment) remains the same.

Let me know if you'd like sensitivity analyses (different allocation ratios, longer accrual, non-exponential survival, etc.) or any further clarification!
